# Seroprevalence of Feline Heartworm in Spain: Completing the Epidemiological Puzzle of a Neglected Disease in the Cat

**DOI:** 10.3389/fvets.2022.900371

**Published:** 2022-05-12

**Authors:** José Alberto Montoya-Alonso, Sara Nieves García Rodríguez, Elena Carretón, Iván Rodríguez Escolar, Noelia Costa-Rodríguez, Jorge Isidoro Matos, Rodrigo Morchón

**Affiliations:** ^1^Internal Medicine, Faculty of Veterinary Medicine, Research Institute of Biomedical and Health Sciences (IUIBS), Universidad de Las Palmas de Gran Canaria, Las Palmas de Gran Canaria, Spain; ^2^Zoonotic Infections and One Health Group, Laboratory of Parasitology, Faculty of Pharmacy, University of Salamanca, Campus Miguel Unamuno, Salamanca, Spain

**Keywords:** *Dirofilaria immitis*, epidemiology, seroprevalence, heartworm, dirofilariosis, cats, Spain

## Abstract

Feline heartworm is a vector-borne zoonotic disease caused by *Dirofilaria immitis*. It is a cosmopolitan disease that is continuously expanding. Spain is considered an endemic country; however, although there are many published studies in dogs, feline heartworm has been poorly studied in this country. Thus, the objective was to analyze the exposure to *D. immitis* throughout Spain to complete the epidemiological map in the feline species. For this, 6,588 feline serum samples were analyzed for the presence of *D. immitis* antigens and antibodies against *D. immitis* and *Wolbachia*. The results were analyzed according to sex, age, breed, habitat, origin (owned or shelter cats), presence of clinical signs, use of preventive, location and climatology. The results showed a prevalence of 0.5% and a seroprevalence of 9.4%. The highest antibody seroprevalences were reported in the Canary Islands and the Balearic Islands (19.2 and 16%, respectively), as well as in the autonomous communities located on the Mediterranean coast (9.2–11.2%). Seropositive cats were found in both indoor and outdoor cats, and from 6 months of age. Furthermore, only 5.8% of cats received regular prophylactic treatment. The results show that feline dirofilariasis is widely distributed throughout the national territory and corroborate that, where infected dogs are present, there are cats exposed to the parasite. It is necessary to implement efficient awareness and prophylaxis measures to control the incidence and expansion of feline heartworm in Spain.

## Introduction

Heartworm infection caused by *Dirofilaria immitis* is a well-known vector-borne disease in the canine population. Discovered in the dog in 1,626, in recent years there have been important scientific advances focused on improving diagnosis, treatment and prevention, as well as knowing the changes in its distribution worldwide (1, 2).

Infection in cats is similar to that in dogs, which is through the bite of a culicid mosquito carrying infective larvae (L3). Although cats can be infected, they are less suitable hosts than dogs, so less larvae develop into adults and adult worms have a shorter lifespan. However, infections with even a single worm can have fatal consequences and present high mortality rates in the cat (3, 4). Knowledge about heartworm in the feline species is much more limited than in the dog. This may be due to a multifactorial problem; mainly, due to the lack of epidemiological studies and the difficulty of diagnosis. It is estimated that many heartworm-infected cats are misdiagnosed with asthma or allergic bronchitis (4, 5). In addition, feline heartworm is frequently asymptomatic, and sometimes sudden death may be the only symptom a cat exhibits. Furthermore, these cats are not usually subjected to necropsy, so the cause of death is rarely clarified contributing to the lack of knowledge of the presence and importance of feline heartworm disease (1, 6).

Spain is an endemic country for *D. immitis*. Recent studies have shown that canine heartworm is present throughout the Spanish geography and is expanding toward northern areas traditionally considered free of the disease (7). This expansion has been described in other European countries and heartworm is therefore considered an emerging disease (8, 9). Although the distribution is well studied in the canine species, there is an important lack of knowledge in the cat; in Spain, there are only published epidemiological studies in the Canary Islands, Madrid, Barcelona and Zaragoza (10–14). However, it is estimated that where canine heartworm is present, feline heartworm disease is also present (15, 16). Therefore, the objective of this study was to complete the epidemiological puzzle of feline heartworm in Spain.

## Materials and Methods

### Climatic Characteristics of Spain

The different climates present in Spain have been characterized using the Köppen Climate Classification (17). The varied orography of Spain, as well as its geographical location, in the middle latitudes of the temperate zone of the Northern Hemisphere, is the cause of a remarkable climatic diversity in the country, but for the analysis of this study only the predominant climates in each region have been considered. The temperate with dry or hot summer climate (Csa) covers most of the Iberian Peninsula and the Balearics, occupying ~40% of its surface. This climate is mainly found in Catalonia, Valencian Community, Balearic Islands, Andalusia, Castilla-La Mancha, Madrid, Extremadura as well as the Autonomous Cities of Ceuta and Melilla, and is characterized by hot summers with the average temperature in the warmest month >22°C. The temperate with dry or temperate summer climate (Csb) is the second most common climate in Spain, occurring in ~22% of the territory. Is mainly present in Galicia and Castilla y León. Like the Csa climate, it has a minimum of rainfall in the summer, but summers are mild as do not exceed 22°C on average in the warmest month. The temperate with a dry season and temperate summer climate (Cfb) is the third most common climate in Spain. It is located in the northern region and mainly presented in Asturias, Cantabria, Basque Country, La Rioja, Navarra, and Aragón; this climate prevails with cold, but not freezing winters and cool or hot summers with an average annual temperature variation of ~10°C. Rainfall is abundant and well distributed throughout the year and there are no dry months. Murcia is mainly represented by the cold steppe climate (Bsk); this correspond to a dry climate, characterized by evaporation that exceeds precipitation on average, but is less than potential evaporation; average temperature is <18°C. Finally, the overall climate present in the Canary Islands is subtropical and desert, moderated by the sea and in summer by the trade winds (Subtr).

### Sample Collection

The present study included 6,588 cats from the entire Spanish geography. A total of 128 veterinary clinics and shelters that collected samples between September 2020 and October 2021 participated in this study. Most of the samples came from cats that had presented to the clinic for routine and periodic check-ups. The criteria for inclusion of cats were being over 6 months of age, with no travel history outside their province and no previous history of heartworm infection. A complete record was kept for each animal, including identification (age, sex, breed and habitat), clinical history, and demographic data. In cats from shelters, the age was estimated and the previous history of the animal was unknown. Owners were informed of the objectives of the study and asked for their consent to participate in this survey.

### Assays

Blood samples were collected for prescribed diagnostic purposes or official monitoring studies and were subsequently made available for this study. Blood was placed in serum tubes and centrifuged. Serum was kept at −20°C until testing. Samples were tested for circulating *D. immitis* antigens using a commercial immunochromatographic test kit (Uranotest Dirofilaria^®^, UranoVet SL, Barcelona, Spain) according to the manufacturer's instructions. Furthermore, serological techniques for anti-*D.immitis* and anti-*Wolbachia* antibody detection were used, as described by Morchón et al. (18) with some modifications. In brief, the plates were coated with 0.8 μg of *D. immitis* somatic antigen and recombinant *Wolbachia* surface protein (rWSP). Serum samples were prepared at 1/100 for anti-*D. immitis* serum antibodies and 1/40 for anti-WSP antibody detection. Anti-feline IgG antibody, horseradish peroxidase-labeled (Kirkegaard and Perry Laboratories, Gaithersburg, MD, USA), was applied at 1/4,000 dilution. The optical densities were measured in an Easy- Reader (Bio-Rad Laboratories, Hercules, CA, USA) at 492 nm. Cut-off points of ELISA *D. immitis* 0.8 and ELISA WSP 0.6 were obtained as arithmetic mean optical density ±3 standard deviations of serum of clinically healthy cats. The cats were considered seropositive when anti-*D.immitis* and anti-WSP antibodies presented jointly (10–13, 19).

### Statistical Analysis and Ethical Statement

Data were analyzed using SPSS Base 20.0 software for Windows (SPSS Inc./IBM, Chicago, IL, USA). Pearson's χ^2^-test and Fisher's exact test were performed between two categorical variables. The Bonferroni correction was used for multiple comparisons. The *t*-student/ANOVA test for independent and normally distributed samples was also used for the comparison of means and the Mann-Whitney/Kruskall-Wallis test in independent samples for the comparison of distributions. In all cases, the significance level was established at *p* < 0.05.

For this study, no ethical approvals were required. All blood samples were routinely collected for prescribed diagnostic purposes or official monitoring studies and subsequently made available to this study. The study was carried out in accordance with the current Spanish and European legislation on animal protection.

## Results

**Figure 1:**
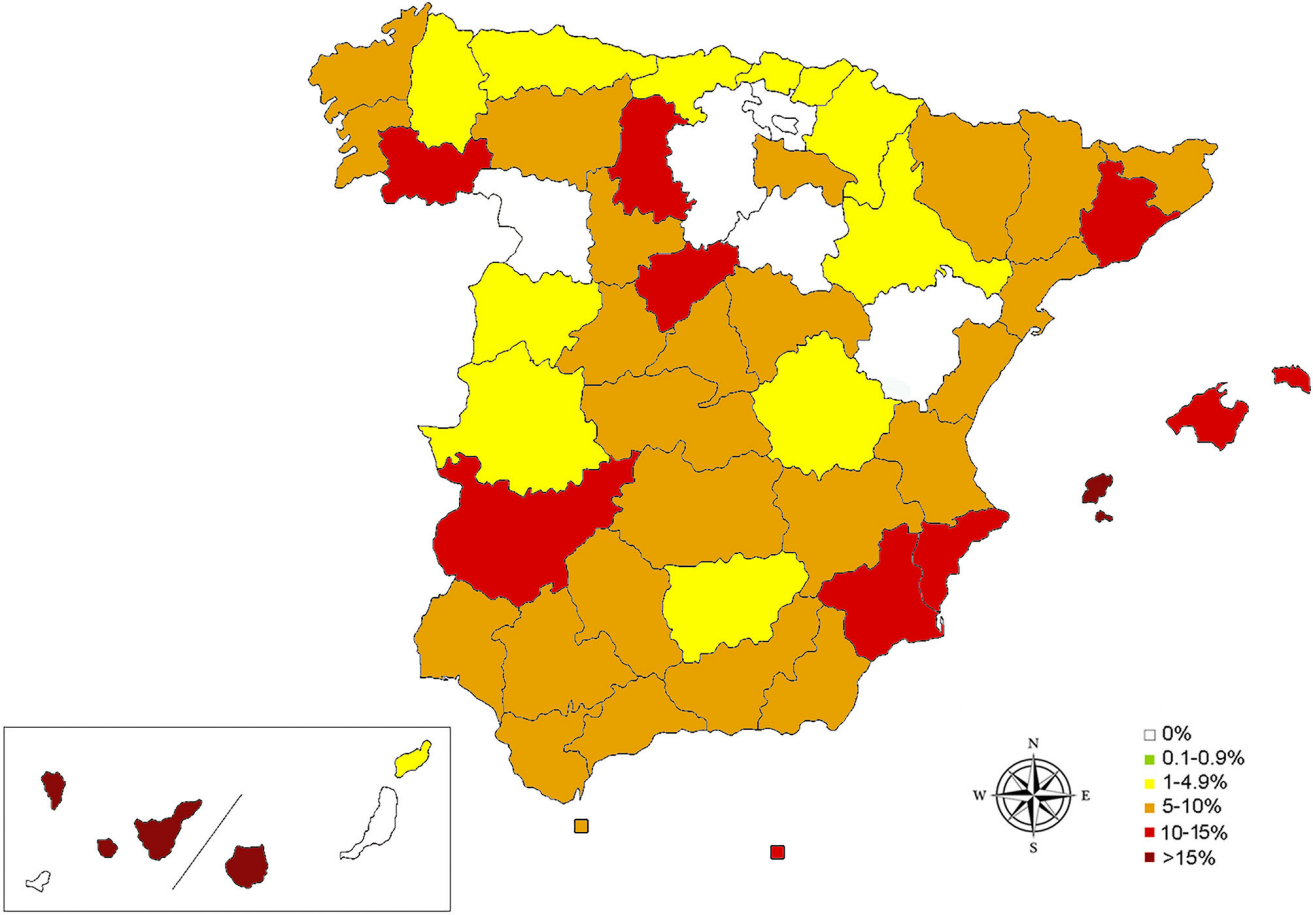
Map of seroprevalences for *D. immitis* in cats in Spain by provinces, islands, and autonomous cities.

The antibody detection test revealed an antibody seroprevalence of 9.4%. Table 1 and Figure 1 shows the result of the prevalence by provinces and autonomous communities. Canary Islands and Balearic Islands showed the highest antibody seroprevalence exceeding 15% (19.2 and 16.0%, respectively). When islands were evaluated separately, the highest antibody seroprevalences were reported on Tenerife (29%), Formentera (28.5%), and Gran Canaria (21.7%). The autonomous communities with the highest antibody seroprevalence in the Iberian Peninsula were Murcia, Catalonia and the Valencian Community (11.2, 10.7, and 9.2%, respectively). When the provinces were evaluated, Badajoz (14.0%), Segovia (12.7%), Alicante (11.8%), Palencia (11.8%), Ourense (11.5%), Barcelona (11.4%), and Murcia (11.2%) exceeded 10% seropositivity.

**Table 1:** Distribution of seroprevalences for *D. immitis* in cats in Spain by autonomous communities, autonomous cities and provinces.

**Autonomous**
**community**	**+/*n* (%)**	**Provinces**	**+/*n* (%)**
Galicia	17/230 (7.4)	A Coruña	5/68 (7.3)
		Lugo	1/47 (2.1)
		Ourense	7/61 (11.5)
		Pontevedra	4/54 (7.4)
Asturias	4/112 (3.6)	Asturias	4/112 (3.6)
Cantabria	3/92 (3.3)	Cantabria	3/92 (3.3)
Basque Country	5/190 (2.6)	Araba	0/20 (0.0)
		Bizkaia	1/37 (2.7)
		Gipuzkoa	4/133 (3.0)
Navarra	3/68 (4.4)	Navarra	3/68 (4.4)
La Rioja	5/65 (7.7)	La Rioja	5/65 (7.7)
Aragón	16/353 (4.5)	Huesca	8/114 (7.0)
		Zaragoza	8/194 (4.1)
		Teruel	0/45 (0.0)
Catalonia	82/769 (10.7)	Girona	5/56 (8.9)
		Barcelona	71/624 (11.4)
		Tarragona	4/49 (8.2)
		Lleida	2/40 (5.0)
Valencian community	23/250 (9.2)	Castellón	7/77 (9.1)
		Valencia	7/97 (7.2)
		Alicante	9/76 (11.8)
Andalusia	94/1,222 (7.7)	Jaén	1/47 (2.1)
		Almería	4/43 (9.3)
		Córdoba	3/42 (7.1)
		Huelva	6/62 (9.7)
		Granada	5/82 (6.1)
		Cádiz	10/109 (9.2)
		Sevilla	62/779 (8.0)
		Málaga	3/58 (5.2)
Murcia	21/187 (11.2)	Murcia	21/187 (11.2)
Castilla-La Mancha	13/245 (5.3)	Guadalajara	3/60 (5.0)
		Cuenca	4/90 (4.4)
		Toledo	2/32 (6.2)
		Ciudad Real	2/32 (6.2)
		Albacete	2/31 (6.4)
Extremadura	10/120 (8.3)	Cáceres	3/70 (4.3)
		Badajoz	7/50 (14.0)
Madrid	61/867 (7.0)	Madrid	61/867 (7.0)
Castilla y León	32/555 (5.8)	León	3/48 (6.2)
		Zamora	0/22 (0.0)
		Salamanca	6/170 (3.5)
		Valladolid	7/103 (6.8)
		Palencia	6/51 (11.8)
		Burgos	0/23 (0.0)
		Soria	0/18 (0.0)
		Segovia	6/47 (12.7)
		Ávila	4/73 (5.5)
Canary Islands	177/921 (19.2)	Tenerife	46/231 (19.9)
		La Palma	27/174 (15.5)
		La Gomera	9/31 (29.0)
		El Hierro	0/17 (0.0)
		Gran Canaria	94/410 (22.9)
		Lanzarote	1/25 (4.0)
		Fuerteventura	0/33 (0.0)
Balearic Islands	45/282 (16.0)	Mallorca	6/51 (11.8)
		Menorca	4/35 (11.4)
		Ibiza	26/164 (15.9)
		Formentera	9/32 (28.1)
Autonomous Cities		Autonomous Cities	+/n (%)
		Ceuta	1/16 (6.3)
		Melilla	2/19 (10.5)
**Total**	**617/6,588 (9.4)**		

*n, number of cats sampled; +, number of seropositive cats; %, percentage of seropositive cats*.

When sex of the cats was assessed, 49.2% females and 50.8% males were observed. No significant differences were observed in the antibody seroprevalence of the disease according to sex (Table 2).

**Table 2:** Seroprevalences for *D. immitis* in cats in Spain by climates, (Köppen Climate Classification System), according to sex, age, origin, habitat, use of prophylaxis, presence of clinical signs, and FeLV/FIV status.

		**Climate**					
**Demographic/**
**epidemiological**
**factor**		**Bsk +/*n* (%)**	**Cfb +/*n* (%)**	**Csa +/*n* (%)**	**Csb +/*n* (%)**	**Subtr. +/*n* (%)**	**Total +/*n* (%)**
**Sex**	Female	12/93 (12.9)	14/428 (3.3)	151/1,796 (8.4)	26/418 (6.2)	105/507 (20.7)	308/3,242 (9.5)
	Male	9/94 (9.6)	22/452 (4.9)	183/2,019 (9.1)	23/367 (6.3)	72/414 (17.4)	307/3,346 (9.2)
**Age**	<1 year	0/22 (0.0)	4/78 (5.1)	25/436 (5.7)	2/117 (1.7)	11/98 (11.2)	42/751 (5.6)
	1–4 years	5/60 (8.3)	17/419 (4.1)	94/1,116 (8.4)	19/303 (6.3)	83/484 (17.1)	218/2,382 (9.2)
	5–10 years	9/61 (14.8)	12/215 (5.6)	99/1,016 (9.7)	14/188 (7.4)	56/228 (24.6)	190/1,708 (11.1)
	11–15 years	6/33 (18.2)	2/104 (1.9)	67/675 (9.9)	9/120 (7.5)	14/60 (23.3)	98/992 (9.7)
	>15 years	1/11 (9.1)	1/64 (1.6)	49/572 (8.6)	5/57 (8.8)	13/51 (25.5)	69/755 (9.1)
**Origin**	Owned cat	18/165 (10.9)	30/752 (4.0)	259/3,288 (7.9)	29/642 (4.5)	91/539 (16.9)	427/5,386 (7.9)
	Shelter cat	3/22 (13.6)	6/128 (4.7)	75/527 (14.2)	20/143 (14.0)	86/382 (22.5)	190/1,202 (15.8)
**Habitat**	Outdoors	4/44 (9.1)	18/199 (9.0)	105/685 (15.3)	19/221 (8.6)	100/460 (21.7)	246/1,609 (15.3)
	Indoors	11/114 (9.6)	8/546 (1.5)	142/2,395 (5.9)	19/429 (4.4)	30/250 (12.0)	210/3,734 (5.6)
	Indoors/outdoors	6/29 (20.7)	10/135 (7.4)	87/735 (11.8)	11/135 (8.1)	47/211 (22.3)	161/1,245 (12.9)
**Prophylaxis**	No	18/141 (12.8)	30/756 (4.0)	330/3,668 (9.0)	38/655 (5.8)	171/864 (19.8)	587/6,084 (9.6)
	Yes	0/12 (0.0)	6/112 (5.4)	3/138 (2.2)	7/71 (9.9)	5/47 (10.6)	21/380 (5.5)
	Intermittently	3/34 (8.8)	0/12 (0.0)	1/9 (11.1)	4/59 (6.8)	1/10 (10.0)	9/124 (7.3)
**Clinical signs**	No	15/149 (10.1)	26/722 (3.6)	253/3,327 (7.6)	23/605 (3.8)	150/790 (19.0)	467/5,593 (8.3)
	Yes	6/38 (15.8)	10/158 (6.3)	81/488 (16.6)	26/180 (14.4)	27/131 (20.6)	150/995 (15.1)
**FeLV/FIV**	FeLV+/FIV Neg	0/0 (0.0)	4/12 (33.3)	7/38 (18.4)	1/13 (7.7)	10/41 (24.4)	22/104 (22.1)
	FeLV+/FIV+	0/2 (0.0)	0/5 (0.0)	0/10 (0.0)	0/0 (0.0)	3/8 (37.5)	3/25 (12.0)
	FeLV Neg/FIV+	0/3 (0.0)	0/12 (0.0)	2/24 (8.3)	2/7 (28.6)	7/30 (23.3)	11/76 (14.5)
	Neg/Neg	7/32 (21.9)	2/79 (2.5)	8/175 (4.6)	0/20 (0.0)	30/198 (15.2)	47/504 (9.3)
	N/a	14/150 (9.3)	30/772 (3.9)	317/3,568 (8.9)	46/745 (6.2)	127/644 (19.7)	534/5,879 (9.1)
**Total**		**21/187 (11.2)**	**36/880 (4.1)**	**334/3,815 (8.8)**	**49/785 (6.2)**	**177/921 (19.2)**	**617/6,588 (9.4)**

*n, number of cats sampled; +, number of seropositive cats; %, percentage of seropositive cats*.

The mean age of the cats was 6.6 years (±5.6 years). There were significant differences in the age distributions between seropositive and seronegative animals, and the median in positive cats is 1 year older than in negative cats. Seropositive cats were found from 6 months of age to 20 years old. By groups of age, the highest antibody seroprevalence was obtained in cats aged 5–10 years (11.1%), followed by cats aged 11–15 years (9.7%), 1–4 years (9.2%), and >15 years (9.1%). The antibody seroprevalence among the youngest (<1 year) was 5.6%, which corresponds to 4–5% points lower than in other older age groups (*p* < 0.001; Table 2).

Samples were collected from 22 different breeds of cats. The most common breed was the European Shorthair being 89.5% of the cats, followed by the Persian (4.6%) and the Siamese (3%). There were no significant differences between the antibody seroprevalences of the different breeds.

Of the studied cats, 81.8% were client-owned and 18.2% were shelter cats. There were significant differences between the prevalence of owned cats (7.9%) and shelter cats (15.8%), being double the antibody seroprevalence in the latter group (*p* < 0.001).

Based on climate, it was observed that the prevalence of the infection was much higher in the subtropical climate (19.2%), while the Cfb climate was the least prevalent with 4.1%. Table 2 shows the prevalence as well as the results according to socio-demographic factors for each climate.

Cats were further divided based on their habitat: 56.6% of the cats were indoors (cats always kept inside the house), 24.4% were outdoors (those always kept outside the house) and 19% were indoors/outdoors (cats that spent at least 1–50% of their time outdoors). Indoor cats showed the lowest antibody seroprevalence (5.6%), up to 8–10% points less than outdoor cats (15.3%) or indoor/outdoor (12.9%) (*p* < 0.001).

When clinical signs were present, these were divided into respiratory signs (cough, dyspnoea, abnormalities on chest x-ray or during chest auscultation), gastrointestinal signs (emesis, diarrhea, constipation, laboratory or imaging signs of hepatitis, pancreatitis, or intestinal inflammation), renal signs (physical, laboratory, or diagnostic imaging changes consistent with acute or chronic renal failure, glomerulonephritis), signs of infectious diseases (compatible with calicivirus, immunodeficiency, leukemia, panleukopenia, and suspected or confirmed feline infectious peritonitis), urinary signs (urolithiasis, lower urinary tract disease, urethral obstruction), hematological signs (anemia, erythrocytosis, coagulation and white blood cells abnormalities), oncological signs (compatible with carcinoma of squamous cells, lymphoma, facial carcinoma, “chest mass effect”), neurological signs (ataxia, seizures, circling, loss of proprioception or sensation), and other signs (oral problems such as gingivitis, gingivostomatitis, dental extractions; weight loss; lethargy; apathy; ocular problems; lameness; behavior problems). Respiratory signs appeared in 18% of symptomatic cats and gastrointestinal signs in 16%. Symptoms were present in 15% of the studied cats; the antibody seroprevalence among those was double that among cats without clinical signs (15.1 vs. 8.3%) (*p* < 0.001; Table 3).

**Table 3:** Seroprevalences for *D. immitis* in cats in Spain based on presence and nature of clinical signs.

**Clinical category**	+/*n* (%)
Respiratory	34/182 (18.7)
Gastrointestinal	19/154 (12.3)
Renal	23/116 (19.8)
Infectious disease	13/101 (12.9)
Urinary	0/42 (0.0)*
Hematological	3/36 (8.3)
Oncological	6/33 (18.2)
Neurological	1/18 (5.6)*
Others	50/307 (16.3)
**Total**	149/989 (15.1)

*n, number of cats sampled; +, number of seropositive cats; %, percentage of seropositive cats*.

*p <0.05.

When chemoprophylaxis was assessed, 92.3% of the cats did not receive prophylactic treatment with macrocyclic lactones compared to 5.8% that did monthly, and 1.9% received it intermittently. The antibody seroprevalence was significantly lower in patients receiving preventive treatment (5.5%) than in those without any prophylactic treatment (9.6%) (*p* < 0.05). Antibody seroprevalence in cats receiving prophylaxis intermittently (not monthly) was 7.3%.

When the presence or absence of feline leukemia (FeLV) or feline immunodeficiency virus (FIV) was assessed, the viral status of 89.4% of cats was unknown, while 7.5% were negative to both viruses; 1.5% were positive to FeLV, 1.2% were positive to FIV and 0.4% were positive to both. Antibody seroprevalence was higher among FeLV positive (22.1%) than among negative cats (9.3%) and cats of unknown status (9.1%) (*p* = 0.000). Antibody seroprevalence in FIV positive cats was 14.5 and 12.0% in cats positive to both viruses.

The antigen test revealed a prevalence of 0.5% (34/6,588). It was observed that the prevalence depended on age, climate, owned/shelter, habitat, presence/absence of symptoms, and viral status (Table 4), although the low number of positive antigen tests did not allow a reliable statistical study. Taking the antibody test as a reference, the antigen test would have a false negative rate of 96.7% (sensitivity of 3.3%) and a false positive rate of 0.2% (specificity of 99.8%).

**Table 4:** Prevalences for antigens of *D. immitis* in cats in Spain by sex, age, climate, origin, habitat, presence of clinical signs, use of prophylaxis and FeLV/FIV status.

**Demographic/**
**epidemiological factor**	**Variables**	**+/*n* (%)**
Sex	Female	22/3,242 (0.7)
	Male	15/3,346 (0.4)
Age	<1 year	2/751 (0.3)
	1–4 years	9/2,382 (0.4)
	5–10 years	17/1,708 (1.0)
	11–15 years	5/992 (0.5)
	>15 years	1/755 (0.1)
Climate	Bsk	2/187 (1.1)
	Cfb	3/880 (0.3)
	Csa	17/3,815 (0.4)
	Csb	1/785 (0.1)
	Subtr	11/921 (1.2)
Origin	Owned cats	19/5,386 (0.3)
	Shelter cats	15/1,202 (1.3)
Habitat	Outdoors	18/1,604 (1.1)
	Indoors	5/3,734 (0.1)
	Indoors/Outdoors	11/1,245 (0.9)
Clinical signs	No	19/5,593 (0.3)
	Yes	15/995 (1.5)
Use of prophylaxis	No	31/6,084 (0.5)
	Yes	2/380 (0.5)
	Intermittently	1/124 (0.8)
FeLV/FIV	FeLV+/FIV Neg	1/104 (1.0)
	FeLV+/FIV+	1/25 (4.0)
	FeLV Neg/FIV+	5/76 (6.6)
	Neg/Neg	13/504 (2.6)
	N/a	14/5,879 (0.2)
**Total**		**34/6,588 (0.52)**

*n, number of cats sampled; +, number of positive cats; %, percentage of positive cats*.

*The handling editor DT declared a past co-authorship with the author EC*.

## Discussion

This study completes, for the first time, the epidemiological map of feline heartworm in Spain, revealing a high antibody seroprevalence. It is observed that the prevalence showed by the antigen test is significantly lower. It is known that the sensitivity of the detection of antigens in infected cats is low, because these hosts generally harbor a low number of adult worms, or because the symptoms produced by their presence occur when the parasites are in larval stages (20–22). On the other hand, a positive result in the antibody test refers to contact with the parasite, not being able to differentiate between past and present infection. Cats have a natural resistance against the parasite, so in many infections the parasite is effectively neutralized by the feline immune system, but presence of antibodies can remain for an indefinite period of time (23). However, a seropositive cat has undoubtedly been exposed to the parasite and is therefore at risk of infection. Thus, the antibody allows knowing the distribution of this parasite among cats, regardless of whether it was a present or past infection. Undoubtedly, the result obtained shows a wide distribution of this parasite among the feline population, very possibly due to the lack of awareness and knowledge about the presence and severity of this parasite among the feline species.

The map shows the presence of cats with presence of antibodies in all the autonomous communities and autonomous cities, confirming the distribution pattern previously observed in canine prevalence studies and the infection risk studies carried out in Spain (7, 24). Furthermore, the results showed that feline heartworm is present where canine heartworm has been reported and demonstrate that *D. immitis* is expanding from areas considered endemic to regions traditionally considered free of heartworm. Within the Iberian Peninsula, Murcia is the autonomous community with the highest antibody seroprevalence in cats, which is consistent with previous studies that have shown Murcia as the region with the highest risk of infection (24) and with the highest prevalence in dogs (7).

Among other factors that favor its expansion, such as climate change, the expansion of competent vector mosquitoes or the transport of reservoir animals, probably the lack of prophylactic measures in populations of dogs and cats in non-endemic areas is allowing the expansion of this parasite. The highest antibody seroprevalences appear in areas of the southern peninsula and the Mediterranean regions, as well as the Canary Islands, areas traditionally considered endemic or hyperendemic for canine heartworm and which show the highest risk of infection (7, 8, 12, 24). As it is a vector-borne disease, its distribution is influenced by the climate, so that those regions with more favorable climatic conditions allow the proliferation of vector mosquitoes and, therefore, presence of higher prevalences (9, 25, 26). In the subtropical climate, where the Canary Islands are located, high antibody seroprevalences of feline heartworm have previously been reported and the present results show a slight increase in the antibody seroprevalence, from 18.1 to 19.2% (7, 12). In the Cfb climate, traditionally considered free of heartworm, low temperatures could hinder the presence of this parasite; however, climate change and the presence of microclimates with higher winter temperatures in large cities, together with other factors not related to climate, could favor the establishment of heartworm in regions dominated by this climate (13).

No statistically significant differences were found based on sex, as has been published in previous studies (12, 13, 27). Likewise, the breed does not seem to influence antibody seroprevalence either, something that has not been demonstrated in cats but has been shown in dogs (7). The fact that the younger age group of cats has a significantly lower antibody seroprevalence is understandable considering that their exposure to the parasite has been lower. However, groups of cats from 1 year of age showed no significant differences between them, which demonstrates the limited prophylaxis carried out in all age groups. In all cases, prophylaxis should start as soon as possible as indicated in the scientific guidelines of international societies (28, 29); furthermore, in this study antibody detection was found in cats from 6 months of age.

Regarding habitat, the constant or partial access to the outdoors had a decisive influence on the result. This is similar to the results obtained in other studies, supposedly due to a greater chance of exposure to vector mosquito bites (10–13, 25, 26). However, seropositive samples were obtained in indoor cats, which highlights the need to take prophylactic measures in all cats, regardless of their habitat.

The percentage of seropositivity in shelter cats was significantly higher. This is probably due to the conditions in which these animals are collected and kept. Generally, these are cats that live or have lived outdoors forming colonies, in insufficient hygienic and food conditions and no prophylaxis against infectious diseases.

Similarly, in this study it is observed that the antibody seroprevalence of *D. immitis* in FeLV positive cats is significantly higher. These results contradict previous publications, which indicate a lack of relationship between positivity to feline viruses and the presence of heartworm (3, 15, 30). Although it could be theorized that the immunosuppression caused by these feline pathologies could favor *D. immitis* infection, it is necessary to carry out more studies to confirm this. In the present study, many of these cats came from shelters, so the double heartworm/FeLV infection could be circumstantial due to the poor conditions in which the cats were living.

Most cats presented to veterinary clinics with diverse clinical signs showed significantly higher prevalences. It has been reported that feline heartworm disease should be added to the differential diagnosis of cats presenting at the veterinary clinic with respiratory and gastrointestinal signs, especially in endemic areas (4). The reason for the high antibody seroprevalence in cats that present other clinical signs, such as oncological or renal signs, is unknown. One possible explanation is that the division into clinical categories may have been imprecise, leading to some distortion of the data. Given this disparity in the results, future studies should be carried out evaluating the clinical signs individually, together with the presence of comorbidities.

Only 5.8% of the cats received prophylactic treatment regularly. As previously mentioned, the lack of prophylactic measures is one of the main problems faced in the fight against this parasite in the feline species. Undoubtedly, the lack of epidemiological studies that demonstrate its distribution as well as the difficulty in diagnosing this pathology in the cat has greatly influenced this lack of knowledge. Logically, the percentage of seropositivity among cats that do not receive prophylaxis is significantly higher than the antibody seroprevalence obtained in cats receiving prophylaxis monthly. The cause of seropositivity in the latter could be due to the incorrect application of prophylactic measures or a false positive result. It could also be caused by an infection prior to starting prophylaxis. Unlike in dogs, it is not common in cats to perform heartworm tests prior to administration of prophylaxis. Moreover, continued exposure, even with preventive therapy, could result in a positive test (5, 29).

The low number of positive antigen tests did not allow a reliable statistical study; however, the results showed that, as observed in the antibody tests, the prevalences were higher in older cats, living in favorable climates, being outdoors, coming from shelters, as well as among those that showed symptoms and were infected by feline viruses. It should be noted that 0.3% of the positive cats were <1 year old, which reiterates the indication to start prophylaxis as soon as possible.

As a potential limitation of this study, it should also be mentioned that a sampling bias may be present. The lack of census data in some autonomous communities does not allow knowing if the collection of samples has been carried out in a balanced way. At the level of autonomous communities, Seville may be over-represented in Andalusia, assuming a bias to the prevalence of the autonomous community.

## Conclusion

These data demonstrate for the first time the great extent of feline heartworm in Spain. This was already reported in previous studies of canine heartworm, so the results of this study show that. Indeed, where there are dogs infected by *D. immitis*, there are also cats infected. However, unlike in the canine species, in the cat there is still not enough awareness about its presence and clinical importance, and prophylactic measures are not applied to the same extent as in dogs. These results show that there is a risk of infection among cats throughout the Spanish geography, and therefore the great need to implement awareness and knowledge measures of this disease among owners and clinical veterinarians, based mainly on continued and adequate prophylaxis. Being the first publication carried out at a national level, more studies are necessary to determine the evolution of this parasitosis in cats.

## Data Availability Statement

The raw data supporting the conclusions of this article will be made available by the authors, without undue reservation.

## Ethics Statement

Ethical review and approval was not required for the animal study. All blood samples were routinely collected for prescribed diagnostic purposes or official monitoring studies and subsequently made available to this study. The study was carried out in accordance with the current Spanish and European Legislation on Animal Protection (Royal Decree 53/2013 and 2010/63/UE Directive). Written informed consent was obtained from the owners for the participation of their animals in this study.

## Author Contributions

EC, JM-A, and RM designed the study and wrote the manuscript. All authors performed the fieldwork, collected the data, performed the experiments, participated in the discussion of the results, corrected, read, and approved the final manuscript.

## Funding

The present study was supported by Merck Sharp and Dohme Animal Health, S.L (CN-240/030/152). NC-R and JM were supported by the Grants for the predoctoral training program for researchers program of the Government of the Canary Islands (TESIS2021010010) and JM was supported by the Grants for the financing of predoctoral contracts program of the Universidad de Las Palmas de Gran Canaria (PIFULPGC-2017-CCSALUD-3).

## Conflict of Interest

The authors declare that the research was conducted in the absence of any commercial or financial relationships that could be construed as a potential conflict of interest. The handling editor DT declared a past co-authorship with the author EC.

## Publisher's Note

All claims expressed in this article are solely those of the authors and do not necessarily represent those of their affiliated organizations, or those of the publisher, the editors and the reviewers. Any product that may be evaluated in this article, or claim that may be made by its manufacturer, is not guaranteed or endorsed by the publisher.
